# Biomechanical analysis of a novel plating for intra-articular distal humerus fractures: combined anteromedial and anterolateral plating

**DOI:** 10.1186/s13018-019-1181-2

**Published:** 2019-05-14

**Authors:** Libiao Wei, Ming Ling, Zhiquan An

**Affiliations:** 1 Department of Traumatic Orthopedics Surgery, Shanghai Jiao Tong University Affiliated Sixth People’s Hospital, No. 600 Yishan Road, Shanghai, 200233 People’s Republic of China; 2 Orthopedic Biomechanical Laboratory, Shanghai Jiao Tong University Affiliated Sixth People’s Hospital, No. 600 Yishan Road, Shanghai, 200233 People’s Republic of China

## Abstract

**Purpose:**

The traditional strategy for fixing intra-articular distal humerus fractures is double plating placed in an orthogonal or parallel configuration, based on posterior approach. With a combined medial and lateral approach, a novel configuration of plating (combined anteromedial and anterolateral plating) has been used. In this study, we investigated the biomechanical properties of the novel plating by comparing it with orthogonal plating.

**Methods:**

Based on the 3D morphology of a healthy subject’s humerus, the models of simple intra-articular distal humerus fractures were simulated. Two configurations of plating were applied to fix the models: the novel plating (with one plate anteromedially and the other anterolaterally on distal humerus), and orthogonal plating. Stresses, displacement, and stiffness were simulated and calculated under the conditions of axial compression, rotation torsion, bending torsion, and valgus torsion by using finite element analysis.

**Results:**

In all the conditions, the maximal von Mises stresses of the novel plating are similar to those of orthogonal plating, and the patterns of stress distribution are similar between these two configurations. However, the impact of high stresses was weaker on the novel plating. The maximal displacement of the novel plating is smaller than that of orthogonal plating. The stiffness of the novel plating is superior to that of orthogonal plating, with the improvements of 19.4%, 122.7%, 25.0%, and 54.2% in axial compression, rotation torsion, bending torsion, and valgus torsion, respectively.

**Conclusions:**

The novel plating is stronger than orthogonal plating without increasing stress magnitude when fixing simple intra-articular distal humerus fractures, which makes it a feasible alternative. Further biomechanical and clinical studies are needed for a decisive conclusion.

## Background

The intra-articular distal humerus fractures have always been challenging for orthopedic surgeons. The prognosis of total elbow arthroplasty are proving to be less predictable than those described for the hip and the knee [1, 2], and internal fixation technique is still the preferred surgical option for patients who are young, active, and in want of quality motor function [3, 4]. Many studies have validated the superiority of double plating technique, which consists of two major configurations, the orthogonal and the parallel plating [5–8]. The orthogonal plating is performed by placing one plate medially and the other posterolaterally, and the parallel plating one medially and the other laterally [6]. What is noteworthy is that the surgeries for intra-articular distal humerus fractures are conducted on the basis of posterior approach, which is a long posterior incision combined with olecranon osteotomy or not [6, 7]. Although the posterior approach is widely used and provides the largest surgical vision [9, 10], approach-related complications have been reported [11–14], including nonunion, malunion, and implant prominence with regard to olecranon osteotomy, decreased muscle strength, ulnar nerve dysfunction, etc.

**Fig. 1:**
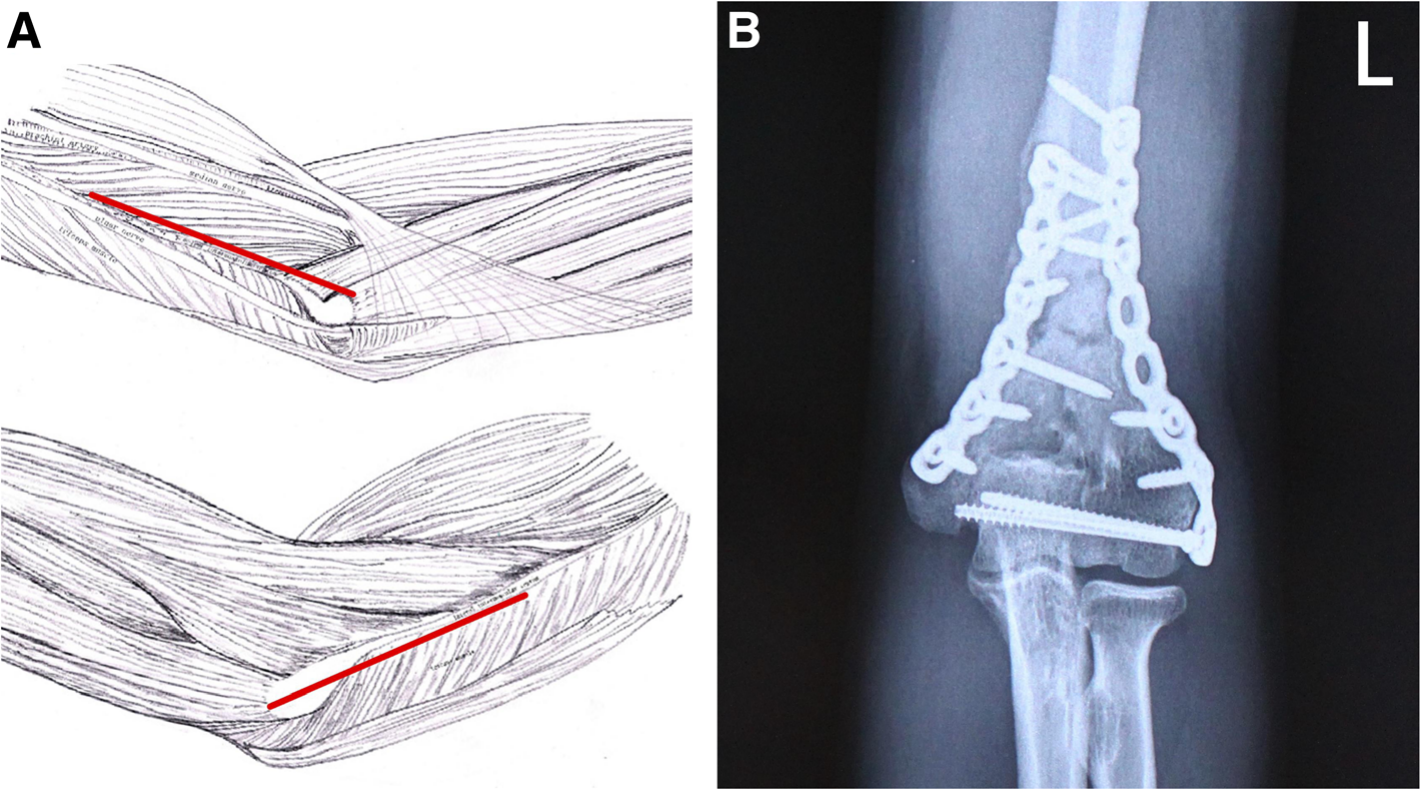
**a** The combined medial and lateral approach. The upper figure shows the ulnar side view, and the lower the radial side view. **b** The X-ray of the novel plating (combined anteromedial and anterolateral plating), with an extra lag screw

The configuration of internal fixation is dictated by sorts of factors, and surgical approach is an essential one of them. In our previous study, we proposed a so-called combined medial and lateral approach for treating intra-articular distal humerus fractures, and a novel configuration with a plate anteromedially and the other anterolaterally came into being accordingly (Fig. 1) [15, 16]. Contrary to previous perceptions, this novel plating locates on the anterior aspect of the distal humerus, which deserves to be further studied.

In this study, we built up the finite element models of the combined anteromedial and anterolateral plating and orthogonal plating, and analyzed their biomechanics. The feasibility of the novel plating was testified compared to orthogonal plating.

## Materials and methods

A computational humerus model was constructed based on the CT scan (Siemens, Germany) data of a 25-year-old male who claimed no history of trauma, pain, and surgery of his left upper arm. Informed consent was signed by him and a pre-diagnostic X-ray was performed before the CT scan. A digital humerus was modeled with meshes about 1 mm. We simulated a simple intra-articular fracture (AO C1 fracture) by segmenting the distal humerus with one fracture line from the middle column to somewhere 5 cm proximal to medial epicondyle, and the other line from olecranon fossae to somewhere 2 cm proximal to lateral epicondyle, resulting in two fragments.

**Fig. 2:**
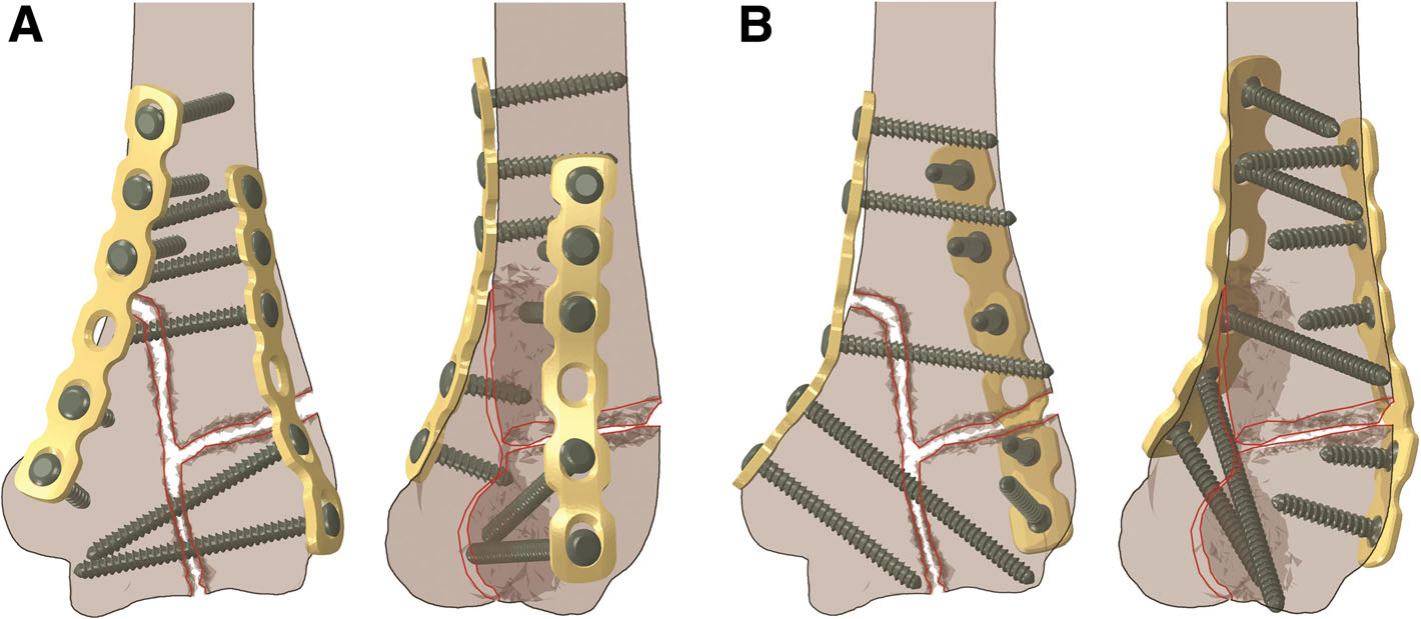
The patterns of the novel plating (**a**) and orthogonal plating (**b**). The left figure shows the front view, and the right the anterolateral oblique view

3.5 mm six-hole reconstruction plates and 3.5 mm diameter full-thread screws were modeled with meshes about 0.5 mm by using the parameters provided by the manufacturer. The novel plating was performed by placing one plate on distal humerus anteromedially and the other anterolaterally, and the orthogonal plating one plate medially and the other posterolaterally (Fig. 2). The plates were contoured to the morphology of distal humerus. Two or three screws were applied on each end of the plates, and the plate holes over or near the fracture lines were left empty. The screws were perpendicular to the plates and with the proper length to ensure bicortical purchase [4, 6].

Depending on the bone segmentation of Mimics 17.0 (Materialise, Belgium), we distinguished the cortical bone from the cancellous bone and assigned them material properties by using the validated expressions of the software (Table 1). The material properties of the plates and screws were in accordance with a previous study (Table 1) [17]. To reduce the computational complexity, all materials were considered elastic without plastic deformity, and C3D4 elements were used for the simulation.

**Table 1:** Material properties of the models

Material	Elastic modulus (MPa)	Poisson’s ratio
Cortical bone	600–21000	0.3
Cancellous bone	207–1401	0.35
Titanium plate	102700	0.34
Titanium screw	110000	0.342

We set up an interaction point which was coincided with the humeral axis and 1 cm above the articular surface. The simulation was conducted under four conditions by applying each of the following loads at the interaction point: (1) axial compression, with 250 N force along the longitude direction and pointing to the proximal, mimicking the condition in which the distal humerus is being compressed; (2) rotation torsion, with 7.5 Nm torque around the longitude direction and rotating inwards, mimicking the condition in which the distal humerus is being twisted inwards; (3) bending torsion, with 10 Nm torque around the coronal direction and rotating backwards, mimicking the condition in which the distal humerus is being bent backwards; (4) valgus torsion, with 10 Nm torque around the sagittal direction and rotating outwards, mimicking the condition in which the distal humerus is being bent outwards. The testing loads were adopted on the basis of the published articles [18–21]. The directions used above were consistent with the CT scan, during which the upper arm was placed in the direction of scanning, flat on, and palm up.

The interactions and boundary conditions were set as the surfaces of the plate holes and those of the half bottom of the screw heads were ‘tied’ to each other, the screws were ‘embedded’ into the bone, the humeral head was fully constrained in all directions and rotations, and the distal articular surface was chosen and applied to the interaction point by using ‘coupling.’

Static simulation was conducted by using finite elements software Abaqus 6.14 (Dassault Systèmes Simulia Corp., USA). We demonstrated the stresses and displacement of each configuration under the described testing conditions. To compare the original data such as maximal stress and displacement, the paired *t* test was used. As to stiffness, which was calculated by dividing the load by the displacement or torque of the interaction point, and of different units in compression and torsion conditions, statistical methods were not used; instead, the comparison was performed by using percentage.

## Results

**Fig. 3:**
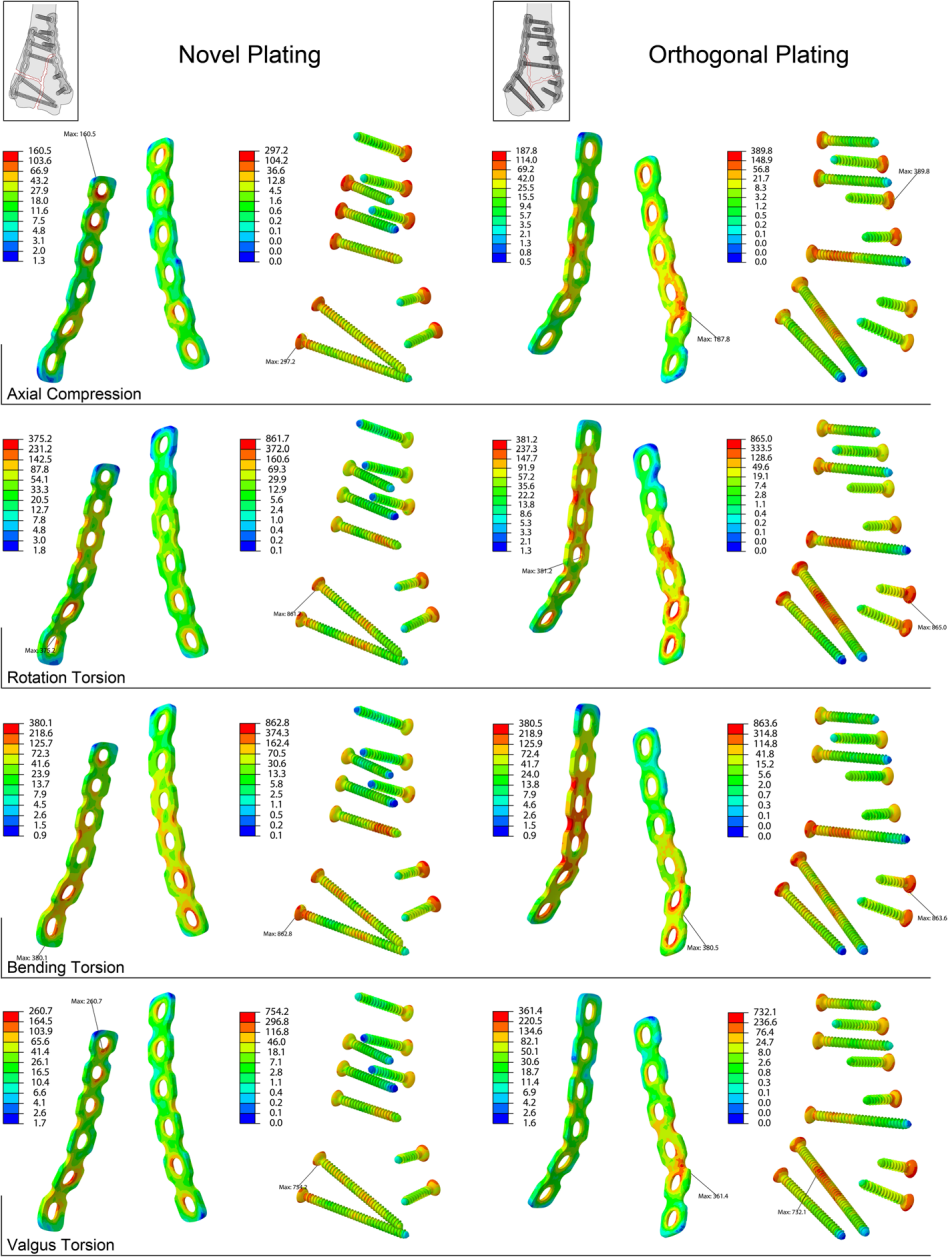
The von Mises stresses of the implants under different testing conditions. For better display effect, the stresses are displayed in log pattern, and the novel plating is demonstrated in rearview, the orthogonal plating in anterolateral oblique view

Plate holes, bending parts of plates, screw heads, and screws bodies across the fracture lines demonstrate relatively high von Mises stresses. It is notable that the impact of these stresses is weaker on the novel plating, compared to orthogonal plating, which means the novel plating is efficient in distributing high stresses. Compared to valgus torsion, bending torsion results in a more prominent distribution of high stresses on both configurations (Fig. 3).

The maximal von Mises stresses occur at plate-screw junctions in most cases. Except that in orthogonal plating, the maximums of plates show up at the bending sites of radial plates in axial compression and valgus torsion, and the maximum of screws shows up at the screw body in valgus torsion (Fig. 3). The maximal von Mises stresses of the bone and implants are shown in Table 2. The maximal stress of bone, plates, and screws between the novel and the orthogonal plating showed no statistical difference in all the testing conditions, with *P* values of 0.992, 0.242, and 0.515, respectively. The maximal stresses of the bone are many times smaller than those of the implants, and they appear mainly around the fracture lines.

**Table 2:** The maximal von Mises stresses of models under different testing conditions

Configuration	Part	Axial compression (MPA)	Rotation torsion (MPA)	Bending torsion (MPA)	Valgus torsion (MPA)
Novel plating	Bone	4.9	32.7	60.4	15.2
	Plate	160.5	375.2	380.1	260.7
	Screw	297.2	861.7	862.8	754.2
Orthogonal plating	Bone	14.4	37.0	33.1	29.1
	Plate	187.8	381.2	380.5	361.4
	Screw	389.8	865.0	863.6	732.1

Larger displacements of the models can be found on orthogonal plating in all the testing conditions (*P* = 0.010), and the largest difference is demonstrated in rotation torsion, in which the maximal displacement of orthogonal plating is twice of novel plating. The values are shown in Table 3. In the conditions of axial compression, bending torsion and valgus torsion, the maximal displacements appear at the distal point of the trochlea; in rotation torsion, that occurs at the medial epicondyle.

**Table 3:** The displacement of models under different testing conditions

Configuration	Axial compression (mm)	Rotation torsion (mm)	Bending torsion (mm)	Valgus torsion (mm)
Novel plating	1.68	0.72	2.08	1.58
Orthogonal plating	2.03	1.43	2.65	1.96

The novel plating demonstrates larger stiffness than orthogonal plating in all the testing conditions, with improvements of 19.4%, 122.7%, 25.0%, and 54.2% in axial compression, rotation torsion, bending torsion, and valgus torsion, respectively (Table 4). The largest difference shows in rotation torsion, in which the stiffness of the novel plating is more than two times of the other. Both configurations are of superior stiffness against valgus torsion.

**Table 4:** The stiffness of models under different testing conditions

Configuration	Axial compression (N/mm)	Rotation torsion (Nmm/deg)	Bending torsion (Nmm/deg)	Valgus torsion (Nmm/deg)
Novel plating	149.4	5549.2	5972.9	12803.3
Orthogonal plating	125.1	2491.5	4779.3	8303.5

## Discussion

There has been a controversy choosing orthogonal plating or parallel plating for intra-articular distal humerus fractures. The AO group recommends orthogonal plating as the first option [6], for it is usually cited as the strongest form of fixation [22, 23]. Besides, the flatness of the posterolateral area of distal humerus reduces the difficulty to place radial plate. However, many more studies support parallel plating for its superior stiffness over orthogonal plating [24–27]. Parallel plating enables more screws to hold articular fragments, which makes it especially suitable for treating low transverse and severely comminuted fractures [28–30].

Different from these two commonly used configurations, the novel plating locates in the ventral aspect of the distal humerus, in which double plating technique is rarely applied. Interestingly, the novel plating demonstrates larger stiffness without raising the stresses in this simulation study. High-stress areas are found at the plate-screw junctions, indicating the transmission of stresses from screws to plates at these areas, thus the maximal stresses always show up there. Apart from that, the bodies of plates and screws are also the key parts to the overall stability. As Fig. 3 shows, high stresses appear at the screw rods across fracture lines and the plate bodies where their shapes are bent. The screws across fracture lines are for integrating adjacent fragments and immobilizing one with the other. By aligning screws with plates, the implants fix all the fragments to plates, then plates to humerus shaft, forming a relatively rigid structure. The bending parts of plates are contoured to the morphology of distal humerus, and they are weak links of the conduction of stresses. As better mechanical properties are preserved by less contouring of the plates, it is probably the reason why the novel plating is of larger stiffness and a more reasonable pattern of stress distribution. The displacement varies in an opposite pattern to stiffness, thus superior overall stability is also demonstrated by the data of displacement. Among the torsion conditions, the novel plating performs the best in valgus torsion, the second in bending torsion, and the worst in rotation torsion. Similarly, orthogonal plating works the worst in rotation torsion, while the circumstance is improved the most by using the novel plating.

Beyond biomechanics, the novel fixation is also feasible in clinical practice. Placing screws through the capitellum-trochlea segment transversely seems to be difficult when using reconstruction plates, due to the protrusion of medial epicondyle. Screws coming from the medial side, more often than not, proceed distolaterally and end between the medial and lateral columns, holding only partial capitellum-trochlea segment (Fig. 2), thus extra use of lag screws would sometimes be complemented. It is notable that the lateral epicondyle curves more slightly than the medial one. Without greatly manual contouring, the radial plate fits the morphology of the lateral column and can be easily placed distally, and it is much easier to insert a transverse screw from the lateral. As seen in Fig. 2, the novel plating contains more distal transverse screws than orthogonal plating. Besides, the anteromedial and anterolateral surfaces of distal humerus shaft provide relatively flat attachment for plates.

The surgical approach, which dictates the intraoperative exposure, is essential for the positioning of plating. Exposing the articular surface by one dorsal incision with or without olecranon osteotomy, the posterior approach has been the golden standard for treating intra-articular distal humerus fractures [6]. Complications have been reported [11–14], but no other approaches present less adverse events and provide an adequate exposure. By applying the combined medial and lateral approach, we reported a 100% good or excellent result among 19 patients undergoing internal fixations due to intra-articular distal humerus fractures, and lower complications rate was reported [16]. The invasive manipulations which are used for further exposure, such as olecranon osteotomy, triceps detachment, and ulna nerve transposition, can be avoided by implementing this approach. As a result, the integrity of ulna is preserved as a reference for better reduction of the distal humerus, and the triceps remains intact to maximize the extension function in the early postoperative stage. Besides, articular surface exposure of 46.9% is discovered in our unpublished study, which can provide sufficient exposure for distal humeral fractures which are less comminuted, such as OA/OTA C1 and C2 fractures.

As with many studies, this computed simulation study has its limitations. Firstly, it may overestimate the magnitude of stiffness, because the fixation could be weakened due to factors such as soft tissue insertion and bone destruction, which were not included in the study. Considering that the factors are inevitable, the relation of stiffness among different testing conditions should stay the same, so it is sufficient to compare the inter-configuration rigidity. Secondly, severely comminuted intra-articular fractures (AO/OTC C3 fracture) were not simulated in the study for extra fixations such as lag screws, headless screws, and K-wires would always be used in these cases [6], making the analysis even more complicated and results unreliable. Thirdly, a larger sample size was not adopted, as we chose a humerus with standard morphology. Besides, large samples size is not that common in finite element analysis.

This study verified the biomechanical feasibility of a novel plating in the treatment of intra-articular distal humeral fractures. The novel plating, which extends the current concepts of the plating configuration, is placed with the usage of double incision instead of the posterior approach, thus avoids some invasive manipulations. The clinical outcome was good in our previous study [16], while further biomechanical and clinical researches are still needed to support it.

## Conclusions

The novel plating (combined anteromedial and anterolateral plating) is feasible for fixing intra-articular distal humerus fractures. When used for simple intra-articular fractures, it demonstrates smaller displacement, larger stiffness, and similar stresses in axial compression, rotation torsion, bending torsion, and valgus torsion, compared with orthogonal plating. The stiffness of the novel plating against rotation torsion improves the most compared with orthogonal plating. Further biomechanical and clinical studies are needed for a decisive conclusion.

## Abbreviations

AO: Arbeitsgemeinschaft für Osteosynthesefragen
CT: Computed tomography
K-wire: Kirschner wire
